# The histone variant H2A.Z in gene regulation

**DOI:** 10.1186/s13072-019-0274-9

**Published:** 2019-06-14

**Authors:** Benedetto Daniele Giaimo, Francesca Ferrante, Andreas Herchenröther, Sandra B. Hake, Tilman Borggrefe

**Affiliations:** 1 Institute of Biochemistry, University of Giessen, Friedrichstrasse 24, 35392 Giessen, Germany; 2 Institute for Genetics, University of Giessen, Heinrich-Buff-Ring 58-62, 35392 Giessen, Germany

**Keywords:** H2A.Z, H2Av, Histone variant, p400, Domino, Tip60, CRISPR/Cas9

## Abstract

The histone variant H2A.Z is involved in several processes such as transcriptional control, DNA repair, regulation of centromeric heterochromatin and, not surprisingly, is implicated in diseases such as cancer. Here, we review the recent developments on H2A.Z focusing on its role in transcriptional activation and repression. H2A.Z, as a replication-independent histone, has been studied in several model organisms and inducible mammalian model systems. Its loading machinery and several modifying enzymes have been recently identified, and some of the long-standing discrepancies in transcriptional activation and/or repression are about to be resolved. The buffering functions of H2A.Z, as supported by genome-wide localization and analyzed in several dynamic systems, are an excellent example of transcriptional control. Posttranslational modifications such as acetylation and ubiquitination of H2A.Z, as well as its specific binding partners, are in our view central players in the control of gene expression. Understanding the key-mechanisms in either turnover or stabilization of H2A.Z-containing nucleosomes as well as defining the H2A.Z interactome will pave the way for therapeutic applications in the future.

## Background

The chromatin structure represents the major modulator of all DNA-based processes such as gene transcription, DNA replication and repair. Chromatin is essentially composed of DNA and histone proteins that together form its basic unit, known as nucleosome. Within the core nucleosome, approximately 146 base pairs (bp) of DNA are wrapped in a left-handed superhelical turn around a protein structure composed of two copies each of the histones H3, H4, H2A and H2B whose crystal structure was solved more than 20 years ago [1]. While histones H3 and H4 form a tetrameric structure known as nucleosome core that is positioned in the inner region of the nucleosome, the histones H2A and H2B are rather located on the nucleosomal surface. In addition, the linker histone H1 can contact the entry and exit sites of the nucleosomal DNA resulting in a more compact structure [2]. Of note, histones are characterized by the presence of a characteristic histone fold domain from which unstructured N- and C-terminal tails protrude [1]. In the process of nucleosome assembly, ATP-dependent remodelers assemble and arrange histone octamers in a highly dynamic fashion [3]. Posttranslational modifications (PTMs) are placed predominantly on flexible histone tails but also within the histone fold domains [4–6]. Importantly, replication-independent (hereafter referred to as non-canonical) histone variants can substitute replication-dependent (hereafter referred to as canonical) histones and are specifically positioned within the genome.

While canonical histones are expressed exclusively during the replication phase of the cell cycle, histone variants are expressed throughout the cell cycle. Canonical histones are encoded by multi-copy genes that lack introns and present a stem loop structure at the 3′-end of their mRNAs. In contrast, genes encoding histone variants are biallelic, sometimes characterized by introns and poly-adenylated at the 3′-end of their mRNAs. As consequence, some histone variants-encoding genes are subjected to alternative splicing. Apart from histone H4, all histone protein families (H2A, H2B, H3 and H1) are characterized by specialized histone variants and among them the most studied family is the H2A, which comprises several members including macroH2A, H2A.X, H2A.Bbd and H2A.Z [7]. As an aside, an H4 variant has been identified in the urochordata *Oikopleura dioica* [8] and in *Trypanosoma brucei* [9], suggesting the possibility that H4 variants may be expressed also in other organisms.

The histone variant H2A.Z has been intensively studied over the last three decades elucidating not only the enzymatic activities required for its chromatin deposition but also the interlinked posttranslational regulatory mechanisms as well as its dynamics in response to signaling pathways. The focus of this review is to summarize and discuss the current knowledge on the histone variant H2A.Z. In particular, we will emphasize the mechanisms of its chromatin deposition and removal, its posttranslational regulation and its interaction partners. Further, we will also review the latest developments concerning H2A.Z’s deregulation or mutations in diseases and how newest technologies can be used to manipulate histone variant levels.

## Historical perspective and overview

The histone variant H2A.Z was originally identified in 1980 in mouse L1210 cells [10]. Few years later, studies in *Tetrahymena thermophila* observed the presence of H2A.Z in the transcriptionally active macronucleus but not in the transcriptionally inactive micronucleus [11]. Later, the *Drosophila melanogaster* homolog H2Av, was identified [12] and shown to be essential [13].

Subsequently, the mammalian *H2A.Z* gene was cloned in 1990 [14] and similarly to *Drosophila*, it was found to be essential since the mouse knockout displays an early-lethal embryonic phenotype [15]. Surprisingly, earlier studies revealed that H2A.Z depletion is not lethal in *Saccharomyces cerevisiae* but “only” leads to reduced growth, a phenotype that can be efficiently rescued via reintroduction of the H2A.Z-encoding gene from *Tetrahymena* [16], marking the evolutionary conservation of H2A.Z.

**Fig. 1:**
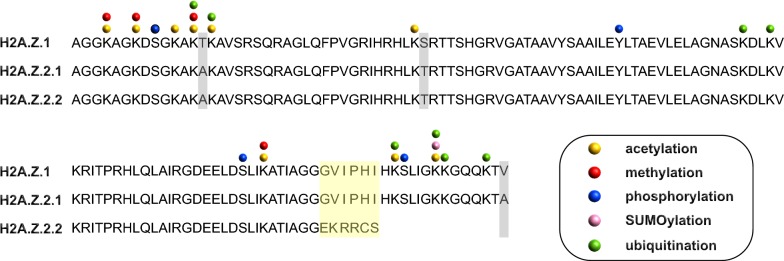
Schematic representation of the different human H2A.Z isoforms. Alignment of H2A.Z.1, H2A.Z.2.1 and H2A.Z.2.2 protein sequences. Highlighted in gray are residues that differ between H2A.Z.1 and H2A.Z.2.1, while residues highlighted in yellow are the ones not conserved between H2A.Z.2.1 and H2A.Z.2.2. Yellow, red, blue, pink and green balls indicate sites of acetylation, methylation, phosphorylation, SUMOylation and ubiquitination, respectively. Please look at Table 2 for more details about the PTMs of H2A.Z

Mass-spectrometry (MS) studies identified two different H2A.Z isoforms that differ only in three amino acids (Fig. 1 [17]). These isoforms, known as H2A.Z.1 and H2A.Z.2 [18], are encoded by two separate genes that are well conserved in chordates and known as *H2AFZ* and *H2AFV* genes, respectively [19]. Even if these two isoforms differ only in three amino acids, they display specialized functions: For example, H2A.Z.2 is preferentially associated with H3K4me3 [20], while H2A.Z.1 has been shown to better interact with the bromodomain-containing protein 2 (BRD2) [21]. Matsuda and colleagues were able to generate single knockouts of both H2A.Z isoforms in chicken DT40 cells further unveiling the different function of the two isoforms. Compared to H2A.Z.1 knockout cells, H2A.Z.2 knockout cells show a slight reduction in proliferation associated with increased apoptosis that may be the consequence of reduced expression of the anti-apoptotic gene *BCL6* [22]. In line with that, H2A.Z.2 depletion in human metastatic melanoma cells leads to downregulation of cell cycle-promoting genes [23] and a recent study further marked the different function of the two isoforms in regulating gene expression in rat neurons [24]. Making use of fluorescence recovery after photobleaching (FRAP) and inverse FRAP (iFRAP), Nishibuchi and colleagues observed that H2A.Z.2 but not H2A.Z.1 is rapidly exchanged at sites of double-strand breaks (DSBs) induced via microirradiation with ultraviolet A (UVA) [25]. Given that RAD51 is required for homologous recombination (HR) at DSBs where it forms foci, the authors investigated the possibility that this mechanism would be differentially influenced by the two H2A.Z isoforms. Surprisingly, they observed reduced RAD51 foci and HR in H2A.Z.2 knockout compared to H2A.Z.1 knockout DT40 cells [25]. However, in the absence of DSBs, another study observed higher mobility of H2A.Z.1 compared to H2A.Z.2 in HeLa cells [26]. Making use of domain swapping experiments, the authors also observed that this difference could be, at least partially, dependent on the substitution in position 38, which corresponds to a serine in H2A.Z.1 and to a threonine in H2A.Z.2 (Fig. 1 [26]).

Recently, this H2A.Z isoform scenario has become more complex due to the identification of an alternatively spliced and primate-specific isoform of H2A.Z.2 (hereafter referred to as H2A.Z.2.1 [18]), known as H2A.Z.2.2 (Fig. 1) that is expressed in a wide range of tissues with maximum transcript expression in human brain tissues [27]. H2A.Z.2.1 and H2A.Z.2.2 differ within their C-terminal region, and H2A.Z.2.2-containing nucleosomes are less stable compared to H2A.Z.2.1-containing ones due to reduced binding to neighboring histones within one octamer [27]. Previously, Adam and colleagues have shown that the C-terminal region of the yeast H2A.Z protein interacts with RNA polymerase II (RNAPII), promoting its recruitment at promoters [28]. Given that the C-terminus of H2A.Z.2.2 significantly differs from the one of H2A.Z.1 and H2A.Z.2.1, it will be interesting to test whether also H2A.Z.2.2 is able to interact with RNAPII and to evaluate whether the different histone variants, present within a different genomic localization, may differentially influence RNAPII recruitment and finally transcription.

H2A.Z has been linked to diverse biological processes such as memory [29–32] and epithelial-to-mesenchymal transition (EMT, [33]). At molecular level, it has been implicated in heterochromatin regulation [34–38], anti-silencing function at boundaries in yeast [39–42], DNA repair [25, 43–48] and transcriptional regulation [21, 23, 28, 29, 49–88]. How H2A.Z regulates such a wide spectrum of different processes is not fully understood, and it is even more surprising that H2A.Z regulates both transcriptional repression and activation.

Interestingly, the *Drosophila* H2Av variant, encoded by the *His2Av* gene, fulfills the functions of both mammalian H2A.Z and H2A.X variants. Similar to mammalian H2A.Z, H2Av regulates heterochromatin formation [89] and gene regulation [90] as also marked by its enrichment in euchromatic regions [91]. On the other hand, the mammalian histone variant H2A.X is a pivotal factor for DNA damage responses. H2A.X is phosphorylated on its unique serine 139 (called γ-H2A.X) upon DSBs and helps recruiting the DNA repair machinery [92]. Similarly, upon DSBs, *Drosophila* H2Av is specifically phosphorylated on a serine residue conserved in mammalian H2A.X [93]. Subsequently, phosphoH2Av is acetylated by the histone acetyltransferase (HAT) dTip60, leading to its exchange with an unmodified H2Av at DSB sites [94]. In line with that, loss-of-function (LoF) of Tip60 leads to accumulation of phosphoH2Av [95].

In this review, we will discuss the recent literature elucidating the contrasting facets of H2A.Z in gene regulation.

## Mechanisms of loading and removal of the histone variant H2A.Z

One major discovery in the histone variant field was the identification of yeast Swr1, a member of the Snf2 family of ATPases, to be the protein complex responsible for loading H2A.Z onto chromatin [62, 63, 98–100]. Swr1 loads the H2A.Z-H2B dimer into nucleosomes by a well-defined mechanism: First, one H2A.Z-H2B dimer is loaded generating an H2A-H2A.Z heterotypic nucleosome, and then a second H2A.Z-H2B dimer is loaded generating a homotypic H2A.Z nucleosome [101]. Swr1 itself is part of the multi-subunit SWR1 complex (Table 1, [63, 96–98, 100, 102]), whose 3D architecture has been recently solved by electron microscopy [103, 104]. Its function is to partially unwrap the DNA from the histone core, which is dynamically altered by ATP consumption [105]. Furthermore, the crystal structure of the central Swr1 enzyme in complex with the H2A.Z-H2B dimer revealed that Swr1 delivers the H2A.Z-H2B dimer to the DNA-(H3-H4) tetrasome as a histone chaperone [106]. Importantly, six of the SWR1 complex subunits are also found within the NuA4 acetyltransferase complex (Table 1) and/or the Ino80 chromatin remodeling complex [97, 98, 100]. The NuA4 complex is a multi-subunit complex [107] involved in the acetylation of H2A.Z by its specific subunit Esa1 [107, 108]. Esa1 and its mammalian homolog, Tip60, are also required to stimulate H2A.Z loading by acetylation of H4 and H2A histone tails within the nucleosome [100, 109, 110]. Mechanistically, H2A/H4 acetylation is required to recruit the SWR1 complex via its subunit Bdf1, which is able to recognize acetylated histones via its bromodomain [100]. In line with this observation, Bdf1 LoF mutants showed reduced H2A.Z chromatin occupancy in yeast cells [82]. Similarly to the NuA4 complex, also the acetyltransferase activity of the something about silencing (SAS) complex (composed of Sas2, Sas4 and Sas5, [111]) is able to stimulate H2A.Z incorporation in yeast [41], further marking that histone acetylation is a prerequisite for H2A.Z deposition.

**Table 1:** Composition of the SWR1, NuA4, Ep400/Tip60, SRCAP and Domino complexes

Yeast		Mammals		*Drosophila*
NuA4	SWR1	Ep400/Tip60	SRCAP	
Tra1		TRRAP		Nipped A
Eaf1	Swr1	Ep400	SRCAP	Domino
	Bdf1	BRD8 (TRCp120)	BRD8 (TRCp120)	Brd8
Epl1		EPC-like (EPC2)		E(Pc)
		EPC1		E(Pc)
Esa1		Tip60		dTip60
Eaf2	Eaf2	DMAP1	DMAP1	Dmap1
Rvb2	Rvb2	Tip49b (Ruvbl2)	Tip49b (Ruvbl2)	Reptin
Rvb1	Rvb1	Tip49a (Ruvbl1)	Tip49a (Ruvbl1)	Pontin
Arp4	Arp4	BAF53a (Actl6a)	BAF53a (Actl6a)	Bap55
Yng2		ING3		Ing3
			ARP6	
Eaf7		MRGBP		Mrgbp
Act1	Act1	Actin		
Eaf3		MRG15 (Morf4l1)		Mrg15
		MRGX (Morf4l2)		Mrg15
	Vps72	YL1	YL1	Yl1
Eaf5		?		
Yaf9	Yaf9	GAS41 (Yeats4)	GAS41 (Yeats4)	dGas41
Eaf6		FLJ11730 (Meaf6; hEaf6)		Eaf6
	Vps71		Znf-HIT1	

The table has been assembled accordingly to the literature [97, 98, 100, 102, 107, 112–115, 210]

*Swr1* is evolutionary conserved: The *Drosophila* homolog is known as Domino, while in mammals there are two homologs called *SRCAP* (*SNF2*-*related CREBBP activator protein*) and *Ep400*, which are both able to catalyze the incorporation of H2A.Z within chromatin [49, 112]. Biochemical purifications of the human Ep400-containing complex surprisingly unveiled that it is composed of not only homologous subunits of the SWR1 complex but also contains subunits that are exclusively found within the yeast NuA4 complex (Table 1, [109, 113–116]). The same is true for the *Drosophila* complex (Table 1, [94]). This suggests that the *Drosophila*/human complex, known as p400/Tip60 complex, represents a physical merge of the yeast SWR1 and NuA4 complexes. This hypothesis is further supported by the observation that human Ep400 represents a fusion of yeast Swr1 and Eaf1, subunits of SWR1 and NuA4 complexes, respectively [107]. In contrast, biochemical purification of the human SRCAP-containing complex showed that it does not contain any histone acetyltransferase activity (Table 1, [112, 116]).

**Fig. 2:**
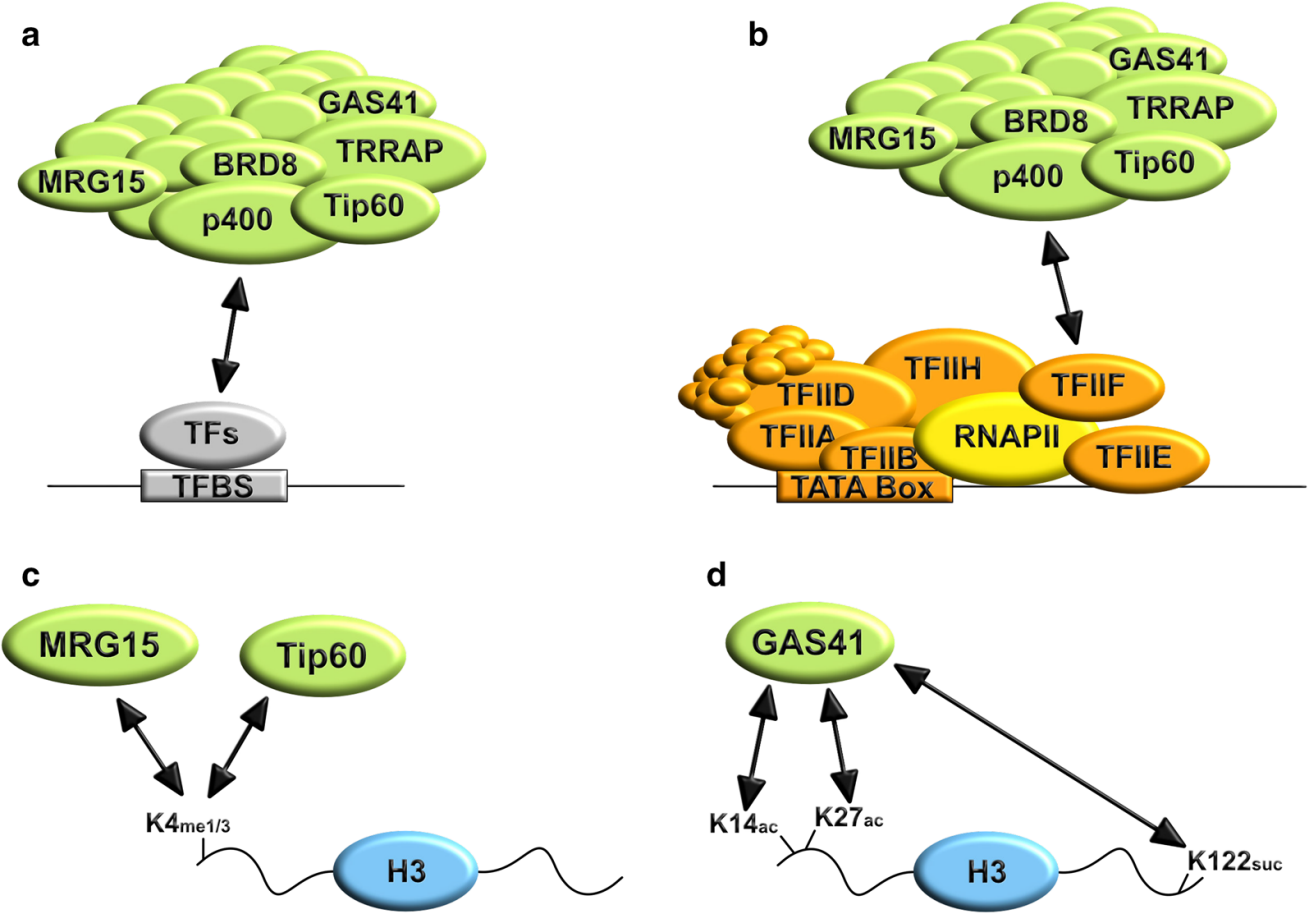
Mechanisms of recruitment of the Ep400/Tip60 complex. The Ep400/Tip60 complex, involved in loading and acetylation of the histone variant H2A.Z, can be recruited to its target genomic sites via interactions with **a** transcription factors (TFs) or **b** subunits of the pre-initiation complex (PIC) composed of general transcription factors (GTF; TFIIA, TFIIB, TFIID, TFIIE, TFIIF and TFIIH) and RNA polymerase II (RNAPII). In addition, the Ep400/Tip60 complex can be recruited to its target genomic sites via interactions with posttranslationally modified histone proteins, for example: **c** MRG15 binds to H3K4me1 or H3K4me3 [122], while Tip60 binds to H3K4me1 via its chromodomain [118]. **d** GAS41 binds to H3K14ac, H3K27ac or H3K122suc using its YEAST domain [123–127]. For simplicity reasons, only the Ep400/Tip60 complex is shown; however, similar mechanisms of recruitment can be used by the SRCAP complex. *TFBS* transcription factor binding site

One important question remains: How is the H2A.Z loading and/or acetylation machinery recruited to chromatin? So far, it was shown that a plethora of different transcription factors (TFs) interact with subunits of the H2A.Z loading complexes (Fig. 2a). For example, the p400/Tip60 complex interacts with the Notch/RBPJ coactivator complex [57], Myc [117], ERα (estrogen receptor alpha, [118]), AR (androgen receptor [119]) and PU.1 [120]. Similarly, the GAS41 (also known as YEATS4) subunit of both p400 and SRCAP complexes interacts with the TFIIF subunit of the pre-initiation complex (PIC, Fig. 2b, [121]). In addition to these interactions, some of the subunits of the p400/Tip60 complex contain “reader” domains able to recognize posttranslationally modified histone tails. For example, MRG15 is able to recognize H3K4me1 and H3K4me3 (Fig. 2c, [122]), Tip60 binds to H3K4me1 (Fig. 2c, [118]) and GAS41, via its YEAST domain, recognizes acetylated or succinylated histone tails (Fig. 2d) in both yeast and human [123–127]. In line with this, GAS41 depletion leads to reduced H2A.Z occupancy [124–126, 128]. It must be also marked that, at least in yeast, the SWR1 complex recognizes a region devoid of nucleosomes, known as nucleosome-free region (NFR) or nucleosome-depleted region (NDR), which is characteristic of transcriptional starting sites (TSSs) [110, 129]. This further suggests an additional mechanism of SWR1 recruitment that involves its interaction with the NFR and that is not mutually exclusive with the mechanisms involving histone PTMs recognition and/or interactions with TFs and/or components of the PIC. However, the involvement of the NFR in the recruitment of the p400/Tip60 and SRCAP complexes has not yet been investigated.

While the SWR1, p400/Tip60 and SRCAP complexes load H2A.Z within chromatin, there are also mechanisms to evict H2A.Z. ANP32E was recently shown to remove H2A.Z from nucleosomes in human cells during DNA damage [130, 131]. Its depletion leads to increased H2A.Z occupancy, and it co-localizes genome-wide with H2A.Z [130–132].

Together, the identification of the protein machineries placing H2A.Z is an important step forward for the better understanding of the dual role of H2A.Z in gene regulation: based on the loading machinery involved in the locus-specific deposition of H2A.Z (SRCAP or p400/Tip60), different PTMs of H2A.Z can be deposited leading to the recruitment of different H2A.Z interactors that finally result in a different transcriptional output (repression or activation).

## Posttranslational regulation of H2A.Z determines the transcriptional output

Histone variants, like all the canonical histones, can be dynamically decorated by various PTMs including acetylation, methylation, phosphorylation, SUMOylation and ubiquitination. Genetic data indicate that H2A.Z can serve as a buffer to quench phenotypic noise via modulating transcriptional efficiencies [133]. Looking at gene expression, H2A.Z depletion can either lead to the upregulation of genes, for example ∆Np63α and Notch signaling targets [29, 49, 57, 72, 134], or to downregulation such as estrogen signaling [28, 50, 54]. Thus, H2A.Z is able to modulate, by dampening, either transcriptional repression or activation. In our view, PTMs of H2A.Z play a major role in this transcriptional buffering function.

Historically, H2A.Z acetylation (H2A.Zac) was first described in *Tetrahymena* [135], while the identification of the exact lysine residues was only fairly recently described (Fig. 1 and Table 2). H2A.Zac is clearly associated with active transcription as first demonstrated in chicken [136, 137]. More recently, these observations have been extended to mammals showing also that H2A.Zac levels positively correlate with transcriptional output [53, 54, 58, 60, 84]. Importantly, H2A.Zac can be dynamically regulated in response to signal transduction [57, 59]. The enzyme responsible for H2A.Zac was initially identified in *Saccharomyces cerevisiae* as Esa1 (Mst1 in *Schizosaccharomyces pombe*), a subunit of the NuA4 complex (Table 1, [40, 99, 108, 138–140]). Subsequently, its homologs have been linked to H2A.Zac also in *Drosophila* and mammals (dTip60 and Tip60, respectively [55, 57, 96, 141]). In yeast, acetylation of H2A.Z’s N-terminal lysine residues does not influence its turnover and this PTM is dynamically regulated due to the deacetylase activity of Hda1 [139, 142]. However, how H2A.Z is deacetylated in higher organisms has not been investigated in depth, although global inhibition of histone deacetylases (HDACs) by trichostatin-A (TSA) leads to increased H2A.Zac [30, 53]. Recently, we have shown that the HDAC1/2-containing nucleosome remodeling and deacetylase (NuRD) complex likely participates in H2A.Z deacetylation [143]. Finally, the characterization of H2A.Zac readers is just at the beginning and so far only bromodomain and PHD (plant homeodomain) finger-containing transcription factor (BPTF) protein and *Plasmodium falciparum* GCN5 (*Pf*GCN5) were shown to recognize a specific pattern of H2A.Zac. BPTF binds to H2A.ZK4acK11ac and H2A.ZK4acK7ac, whereas *Pf*GCN5 interacts with H2A.ZK4acK11ac [144].

**Table 2:**
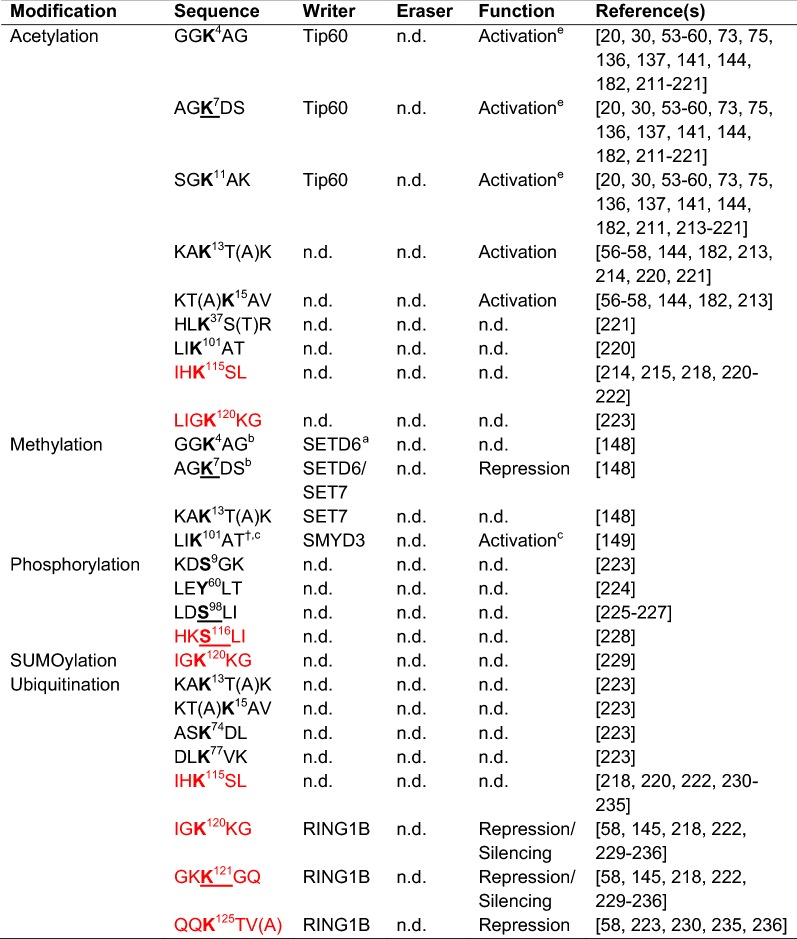
Posttranslation modifications (PTMs) identified in human, mouse and/or rat histone variant H2A.Z

^a^Partially; ^b^monomethylation; ^c^dimethylation; ^d^only referred to dimethylation; ^e^in the case of interferon gene expression, this PTM seems to be associated with a poised/repressed state [73]

PTMs are listed without discriminating among the different H2A.Z isoforms. Residues shown in bold are the modified ones; the residues specific for H2A.Z.2 are shown in parenthesis; shown in red are the residues that are not conserved in H2A.Z.2.2. Underlined are residues subjected to posttranslational regulation that are mutated in diseases accordingly to the COSMIC database. n.d.: not determined. Please look at Fig. 1 for further details about the protein sequences

Ubiquitination of H2A.Z (H2A.Zub) occurs on different lysine residues as summarized in Fig. 1 and Table 2. However, the function of only few ubiquitinated lysine residues has been described. Sarcinella and colleagues observed ubiquitination of H2A.Z on K120 and K121 and linked these modifications to X-chromosome inactivation (XCI) [145]. K120, K121 and K125 monoubiquitination (K120ub1, K121ub1 and K125ub1, respectively) is mediated by RING1B [58, 145]. Active H2A.Z deubiquitination, mediated by USP10 (Ubiquitin-Specific Protease 10), is required to induce gene expression [51]. Furthermore, RNF168 ubiquitinates H2A.Z, but the exact target lysine is still unknown [146]. Surprisingly, Ku and colleagues observed in mouse embryonic stem cells (mESCs) that a fraction of H2A.Zub1 is also acetylated on its N-terminal tail: This population is more acetylated and contains a differential acetylation profile compared to the non-ubiquitinated H2A.Z [58]. It still needs to be investigated whether such a dually modified H2A.Z is an exclusive feature of mESCs or does also occur in other cell types.

The small ubiquitin-like modifier (SUMO) is another member of the ubiquitin peptide family (Fig. 1 and Table 2). SUMOylation of H2A.Z (H2A.Zsu) in yeast has been linked to DNA repair, as it is required for the recruitment of DSBs to the nuclear periphery [43]. Similarly, in HeLa cells, H2A.Z.2su by the SUMO E3 ligase PIAS4 is involved in DNA repair [147], but the exact site modified by PIAS4 has not yet been identified.

In the last years, also H2A.Z methylation was identified which, based on methylation state and the specific lysine residue to be modified, can have different transcriptional outputs (Fig. 1 and Table 2). Monomethylation of lysine 7 of H2A.Z (H2A.ZK7me1), mediated by SETD6, is associated with gene repression in mESCs [148], while dimethylation of lysine 101 (H2A.ZK101me2) is linked to gene induction in human cells [149].

Together, like the PTMs of canonical histones, there is a complicated network of activating and repressing marks also for H2A.Z. However, there are few valuable marks that will, in our view, pave the way for unraveling the molecular mechanisms of H2A.Z in gene regulation.

## The H2A.Z interactome

Assuming that placement of H2A.Z and PTMs of H2A.Z are read and interpreted, it is important to first define the “H2A.Z interactome”. The working hypothesis is interacting factors will give decisive insights about the molecular mechanisms conducting either gene activation or repression.

In the past, several studies examined the H2A.Z interactome using varying methods, such as affinity purification of H2A.Z in nuclear extracts, of either recombinant H2A.Z-containing nucleosomes [150] or H2A.Z mononucleosomes prepared by micrococcal nuclease (MNase) digestion followed by (quantitative) MS and bait protein–protein interaction-sequencing (bPPI-seq) [21, 109, 130, 151, 152]. The results of these studies are summarized in Table 3. Most likely, due to the use of distinctive approaches, different H2A.Z interactors have been identified. Affinity purification of nuclear, not chromatin-bound H2A.Z allowed the identification of H2A.Z-specific chaperone/remodeling complexes (e.g., p400/Tip60, SRCAP complexes, ANP32E and MBTD1) [130]; however, many chromatin-associated proteins remained, most likely, insoluble under the mild conditions used. Consequently, immunoprecipitation of H2A.Z-containing mononucleosomes obtained via MNase digestion of chromatin (MNase-IP) led to the identification of chromatin-bound factors or even large complexes, which stably interact with intact H2A.Z nucleosomes. Nevertheless, this method is entirely restricted to chromatin regions that are accessible for MNase digestion and does not consider strongly compacted and MNase-inaccessible regions. While both of these assays depend on the protein purification quality, bPPi-seq does not. It depends on the formation of a functional eGFP fluorescent protein, when the respective N- and C-terminal parts of eGFP, fused to two proteins, come together in a physical interaction scenario. Hence, bPPi allows the identification of possible direct interactors, meanwhile being limited in identifying large complexes of interactors as the formation of eGFP is dependent on the proximity and the correct steric orientation of the associated factors.

**Table 3:**
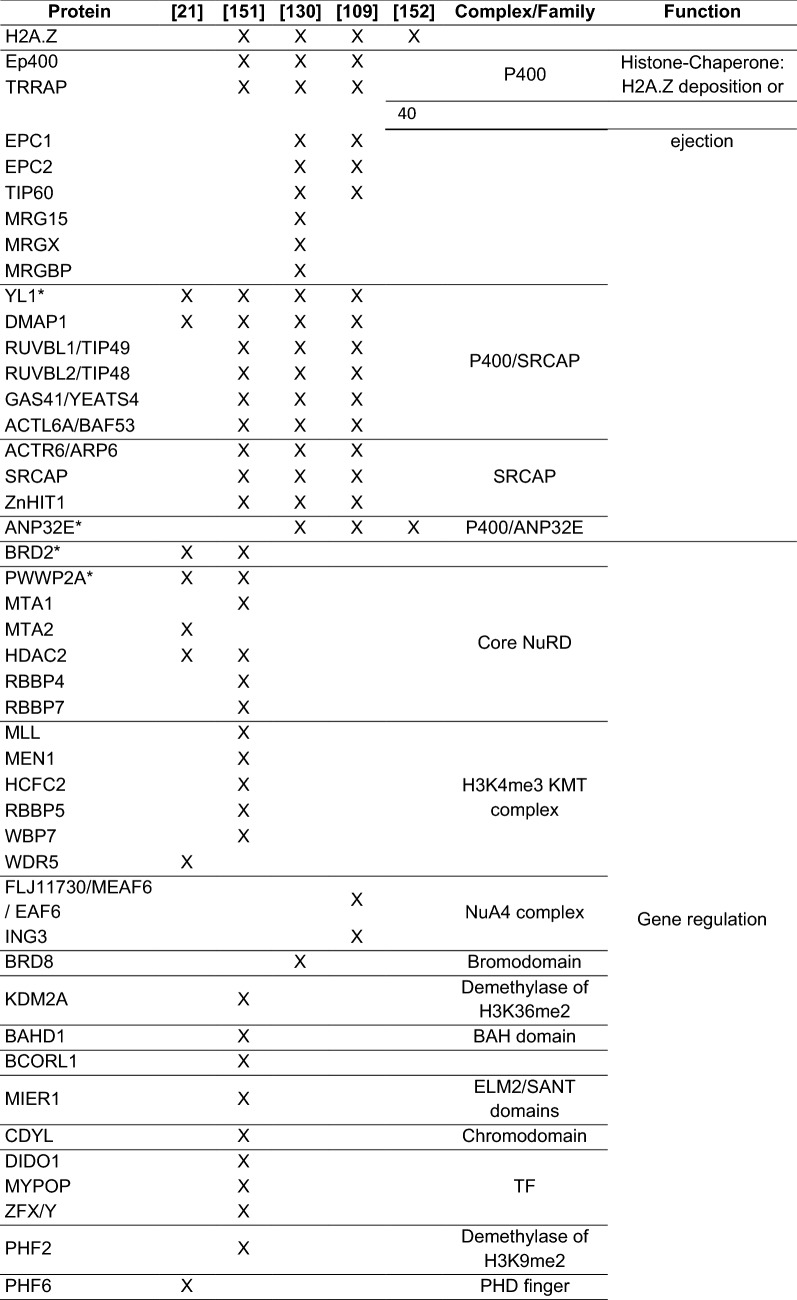

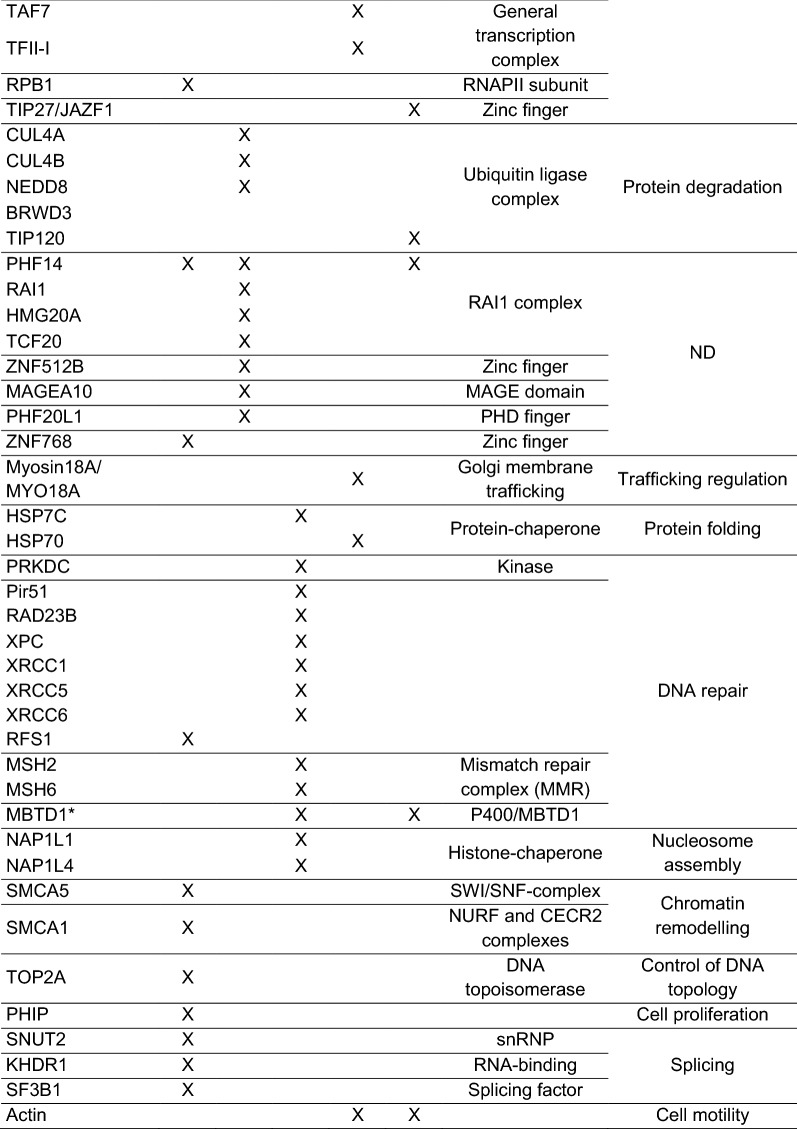
H2A.Z nucleosome-associated proteins

Asterisks indicate nucleosomal H2A.Z interactors validated by immunoprecipitations followed by western blot [21, 109, 130, 151, 152]

Two of the many found H2AZ interactors (Table 3) were biochemically verified and functionally characterized. The first one is BRD2 that was identified by affinity purification of MNase-digested chromatin as an H2A.Z binder on chromatin level [21]. Further, BRD2 was proposed to be a decisive downstream mediator that couples H2A.Z to AR-induced gene activation [21]. It binds H2A.Z-containing nucleosomes via its bromodomains promoted by H4 hyperacetylation and prefers, mediated by a so far unknown mechanism, binding to the H2A.Z.1 over the H2A.Z.2 isoform [21, 144]. Strikingly, H2A.Z.2 was shown to promote and/or maintain BRD2, E2F1 and histone acetylation levels in malignant melanoma [23]. H2A.Z.2 recruits BRD2 and E2Fs, along with HAT activity, to promoters of E2F target genes in melanoma cells, facilitating expression of cell cycle genes and, ultimately, promoting cell proliferation. The other, recently identified protein is PWWP2A that was shown to tightly bind H2A.Z via a multivalent binding mode [151]. PWWP2A’s direct binding to H2A.Z is predominantly mediated by a C-terminal section of its internal protein region of no known homology or structure. Real-time-lapse microscopy imaging showed halt of PWWP2A-depleted cells in mitosis for up to 10 h. A similar effect has been observed in H2A.Z double knockout vertebrate cells [153]. Hence, PWWP2A might be the mediator of the H2A.Z-dependent cell cycle progression phenotype. Interestingly, PWWP2A, as well as H2A.Z, interacts with an MTA1-specific subcomplex of the NuRD complex that was named “M1HR” [143]. This subcomplex consists exclusively of MTA1, RBBP4/7 and HDAC2 and excludes CHD, GATAD2 and MBD proteins. Depletion of PWWP2A increased acetylation of histones in a subset of H2A.Z-containing enhancers bound by PWWP2A where it presumably regulates histone acetylation levels via M1HR recruitment.

Furthermore, H2A.Z was shown to interact with components of complexes involved in a multitude of biological processes, for example DNA damage repair (e.g., MSH2 and MSH6 of the mismatch repair complex, as well as PIR51, RAD23B and XPC), gene activation (e.g., MLL/KMT complex, PHF2, BRD8, MEAF6, ING3), gene repression (e.g., TIP27/JAZF1, BAHD1, BCORL1, MIER1 and CDYL), various transcription factors (e.g., DIDO1, MYPOP, ZFX/Y), chromatin remodeling (e.g., SMCA1 of the nucleosome remodeling factor (NuRF) complex) and proteins whose function(s) remain yet elusive like the RAI1 complex [154, 155], ZNF512B, MAGEA10, PHF20L1 and ZNF768.

Besides the further need to validate all mentioned putative H2A.Z interaction partners in independent biochemical and functional assays, it is tempting to speculate that these many interactors are one important reason why H2A.Z bears transcriptional activating as well as repressing features. At the same time, it shows that although a lot about H2A.Z’s interactome was resolved, its role in recruiting transcription-regulating complexes to their destinations on chromatin still remains a puzzle.

## H2A.Z at enhancers and promoters

In the past, genomic localization of H2A.Z has been mostly reported at the TSS of genes, but more recently it is becoming increasingly clear that H2A.Z is also found at enhancers. In yeast, H2A.Z is strongly enriched at the TSS of both active and inactive genes [156]. Its occupancy at the TSS negatively correlates with gene expression: H2A.Z occupancy is more pronounced at poorly expressed genes compared to induced genes [61–64]. In contrast, genome-wide studies in human CD4^+^ T cells observed H2A.Z enrichment mainly at the TSS of active genes [157, 158]. Subsequently, this scenario was further refined with the observation that H2A.Z occupancy at TSS correlates with the level of transcriptional output: While Ku and colleagues observed a negative correlation [58], other studies observed a positive correlation between gene expression and H2A.Z occupancy [60, 67, 84]. Furthermore, usage of dynamic systems showed that gene induction is associated with reduced H2A.Z occupancy at TSS [54, 68, 70–72] as well as at enhancers [49, 54, 57, 69, 71, 72]. Similarly, in *Drosophila* as well as in plants, H2A.Z occupies the promoter in absence of gene expression but it decreases upon gene induction [65, 66]. Notably, H2A.Z occupancy strongly correlates with H3K4 methylation states [58, 74, 157, 159–161], further marking its involvement in gene poising and activation. The inverse correlation between H2A.Z occupancy and transcription is also reflected in RNAPII occupancy, [62]: H2A.Z is actively excluded from coding regions by the RNAPII-associated remodelers FACT (facilitates chromatin transcription) and spt6 [162]. Deletion of *spt16*, a gene encoding a FACT subunit, or of *spt6,* leads to H2A.Z accumulation at coding regions, a phenotype associated with increased cryptic transcription [162]. This is in line with the observation that H2A.Z-containing nucleosomes are not enriched with H3K36me3, a histone mark associated with transcriptional elongation [143, 159] and provide a mechanistic explanation to the increased H2A.Z occupancy observed at coding regions upon reduced transcription [67, 76, 77]. Additionally, H2A.Z knockdown leads to reduced RNAPII recruitment at TSSs in *Saccharomyces cerevisiae* and human cells [28, 76] and reduced TBP (TATA-binding protein) occupancy *in Saccharomyces cerevisiae* [66]. However, it plays a positive function in preventing RNAPII stalling, as its depletion increases this phenomenon [78, 79], further marking the strong relationship between H2A.Z and RNAPII. Increased cryptic transcription observed at coding regions upon H2A.Z accumulation in yeast [162] would suggest the involvement of H2A.Z in promoter usage, but it must be noted that, at least in human cells, H2A.Z is strongly enriched at facultative heterochromatin without leading to cryptic transcription [76]. A further indication that H2A.Z may be involved in promoter usage is represented by the observation that dispersed core promoters (promoters in which the TSS spreads over hundreds of nucleotides) show a stronger H2A.Z enrichment compared to focused core promoters (promoters in which the TSS occurs in a narrow genomic window of few nucleotides [164]). This dispersion in promoter usage may be the consequence of a different stability of H2A.Z-containing nucleosomes [163], an aspect that will be further discussed in the next section. To note, not only H2A.Z enrichment but also the proximity of H2A.Z-containing nucleosomes to the TSSs influences gene expression [80].

As previously marked, the first genome-wide studies of H2A.Z described its strong enrichment near the TSSs in yeast, *Drosophila* and human [62, 81, 82, 156–158, 164, 165]. However, in subsequent studies it became increasingly clear that H2A.Z is not exclusively found at TSSs but it can also be detected at other regulatory elements such as enhancers and insulators in several different species, though to a lesser extent [58, 83–86, 143, 166]. For example, H2A.Z is enriched at a p53-binding site located approximately 2.2 kb upstream of the TSS of the human *p21* gene [49], further suggesting H2A.Z as an enhancer mark. In line with that, H2A.Z is enriched at an ER binding site located just upstream of the TSS of the human *TFF1* gene, where it localizes in an estrogen-dependent fashion [50]. Genome-wide studies observed a stronger binding of ERα to its genomic sites associated with H2A.Z compared to those sites depleted of H2A.Z [87]. Additionally, at ER sites enriched for H2A.Z, this histone variant seems to be required for the recruitment of RNAPII, for the induction of enhancer RNAs (eRNAs) and finally for the recruitment of RAD21 that is involved in chromatin looping [87]. Similarly, H2A.Z is enriched at AR responsive enhancer sites such as the ones of the *prostate specific antigen* (*PSA*) and *kallikrein*-*like 2* (*KLK2*) genes [51, 52] as well as at the enhancer site of *MyoD* [56]. Recently, we have extended these observations also to developmental signaling pathways: H2A.Z localizes at Notch responsive enhancers where it plays a negative role with regards to the expression of Notch target genes [57]. Interestingly, it has been observed that pioneering factors-bound enhancers exist with two different H2A.Z distributions: a) H2A.Z localizes at the center of Ets1- and Oct4-bound enhancer sites, whereas b) it is enriched at nucleosomes flanking forkhead box protein A2 (FoxA2) or C/EBPα-bound enhancer sites [167]. Table 4 provides the complete list of enhancers, which are bound by signal-specific TFs and have been linked to H2A.Z-mediated regulation.

**Table 4:** Histone variant H2A.Z at enhancers

TFs	Effects of H2A.Z depletion	References
AhR	Reduced induction upon treatment	[88]
AP-1	Upregulation	[72]
AR	Reduced induction upon treatment	[51, 52, 59, 119]
ER	Lack of induction upon treatment	[50, 54, 87, 118]
FoxA2	n.d.	[237]
GR	n.d.	[69, 86]
ISGF3	Increased induction upon treatment	[73]
Muscle differentiation^b^	n.d.	[56]
Myc	n.d.	[77]
p53	Upregulation^a^	[49, 134, 217]
PU.1	n.d.	[120]
∆Np63α	Upregulation	[134, 217]
RARγ	n.d.	[71]
RBPJ	Upregulation	[57]
SMAD3	Downregulation	[161]

^a^Variable based on the cell type; ^b^the TFs have not been investigated; however, H2A.Z localizes at enhancers

The table lists only the cases described in mouse and human. Enhancers are defined as those sites that are bound by signal-specific transcription factors (TFs). *AhR* aryl hydrocarbon receptor (also known as dioxin receptor), *AP*-*1* activator protein-1, *AR* androgen receptor, *ER* estrogen receptor, *FoxA2* forkhead box protein A2, *GR* glucocorticoid receptor, *ISGF3* interferon-stimulated gene factor complex 3 (IRF9 and phosphorylated STAT1 and STAT2), *RARγ* retinoic acid receptor γ, *n.d.* not determined

## H2A.Z and the nucleosome-free regions (NFRs)

The TSS of active genes was previously known as a nucleosome-free region (NFR) or nucleosome-depleted region (NDR). Interestingly, this has been challenged by Jin and Felsenfeld in 2007 [168]. In this study, the authors observed that nucleosomes containing both H2A.Z and H3.3 histone variants are highly unstable and found at regulatory regions such as promoters and enhancers [168]. When nucleosomes are isolated at low salt concentrations, it is possible to observe occupancy of H3.3/H2A.Z double-containing nucleosomes at NFRs, an occupancy that is lost when high salt concentrations are used [83], further suggesting that NFRs might indeed be not nucleosome-free [169]. Such unstable nucleosomes are also enriched at NFRs in *Drosophila* and yeast [170, 171]; however, the reason why the H3.3/H2A.Z double-containing nucleosomes are highly unstable remains unclear. While H2A.Z and the canonical H2A differ significantly in their L1 loop [172], cell-free studies observed that nucleosomes composed of both H2A and H2A.Z (defined as heterotypic, [173]) are more stable than H2A.Z only-containing nucleosomes (defined as homotypic), which are less stable than H2A homotypic nucleosomes [174, 175]. However, a highly unstable heterotypic nucleosome occupies the TSS in the G1 phase of the cell cycle [176, 177]. Furthermore, it should be noted that cell-free studies observed that H3.3 does not alter the stability of H2A.Z both homo- and heterotypic nucleosomes [175, 178]. Based on these data, one could think that neither the incorporation of H3.3 into an H2A.Z-containing nucleosome or the presence of an H2A.Z/H2A heterotypic nucleosome could be responsible for the nucleosome instability observed at NFRs; however, the different approaches used, in vitro (cells) versus cell-free assays, may lead to discrepancies and actually the cell-free approaches may lead to underestimate the nucleosome instability as consequence of the lack of PTMs and/or interactors that may contribute to the regulation of nucleosome stability in a physiological context. It is possible that such nucleosomes would be H2A.Z homotypic. However, at least in *Drosophila*, homotypic H2A.Z nucleosomes are not enriched at the TSS [179], excluding this possibility. In contrast, two more studies observed increased stability of the H2A.Z-containing nucleosomes compared to the H2A-containing nucleosomes in cell-free assays [180, 181]. It seems that this increased stability can be counteracted by histone acetylation, including H2A.Zac [181]. In more detail, it appears that H2A.Zac is the key modification that destabilizes the nucleosome and that acetylation of other histone proteins alone is not sufficient to achieve this destabilization; even more, heterotypic nucleosomes are destabilized by H2A.Zac [182]. While the previous studies focused on H2A.Z.1, another study found structural differences in the L1 loop when comparing this isoform with H2A.Z.2.1 [26]. Furthermore, H2A.Z.2.2-containing nucleosomes seem to be less stable than the H2A.Z.2.1-containing ones [27]. As consequence, at least in vertebrates (or in primates in the case of H2A.Z.2.2), the high instability of the nucleosomes located at NFRs can still be due to the occupancy of the different H2A.Z isoforms that can organize different homo- and heterotypic nucleosomes (also in combination with H3.3) that are regulated by different combinations of PTMs. However, this remains a hypothesis that needs to be tested to identify the mechanism(s) how the nucleosomes occupying the “NFR” become unstable.

## The role of H2A.Z in nucleosome positioning

**Fig. 3:**
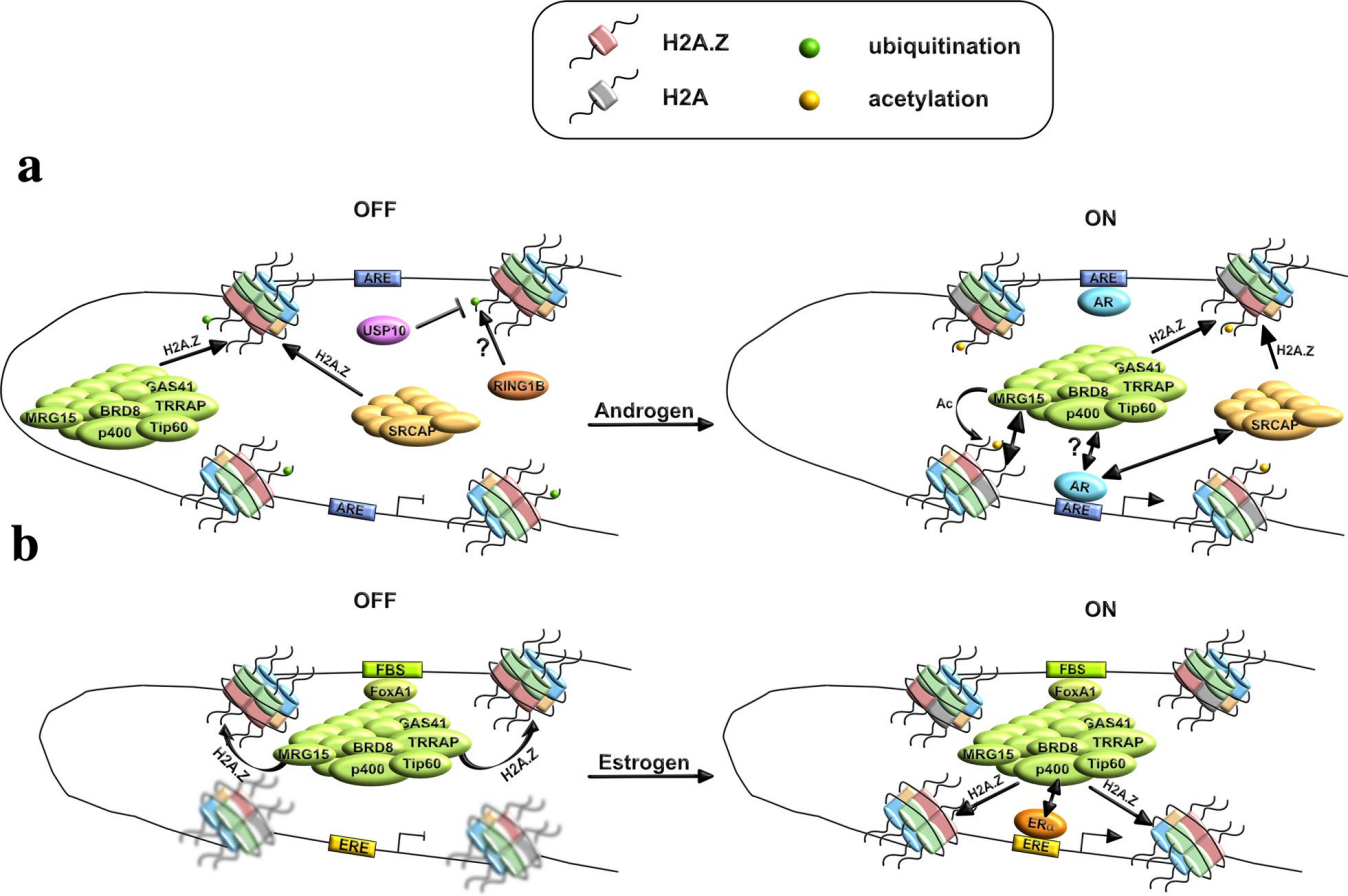
Regulation of H2A.Z and its involvement in transcription. Two examples are used to explain the function of H2A.Z in gene regulation: **a** the case of the androgen system focusing on the *PSA* locus and **b** the case of the estrogen system focusing on the *TFF1* locus. **a** In a repressed or poised (OFF) state, the H2A.Z-specific loading machineries are recruited to the *PSA* locus via not well-defined mechanisms that may involve TFs and/or histone modifications. In this scenario, H2A.Z is deposited by SRCAP and/or p400/Tip60 complexes [119, 122] and the deacetylation and ubiquitination machineries are probably recruited via interactions with DNA binding proteins and/or posttranslationally modified histones (not depicted in the figure). The deacetylation machinery removes the acetylation mark from H2A.Z, which is instead ubiquitinated on its C-terminus by E3 ubiquitin ligases (we speculate that RING1B is involved in the AR signaling cascade [58, 145]). Upon gene activation (ON), deubiquitination (for example, USP10 [51]) and loading/acetylation/deubiquitination machineries are recruited/stabilized via interactions with the Androgen Receptor (AR) that binds to its cognate sequences (androgen receptor-binding element, ARE) and/or histone modifications [119, 122]. This leads to H2A.Z deubiquitination [52] and acetylation [59, 122] finally leading to gene activation which is associated with reduced H2A.Z occupancy [51, 52]. **b** In a repressed (OFF) state, FoxA1 binds to the distal FoxA1-binding site (FBS) of the *TFF1* locus where it recruits the p400/Tip60 complex that supports loading of H2A.Z. In this state, H2A.Z is poorly enriched at the *TFF1* promoter and, as consequence, nucleosome occupancy is poorly defined [50]. Upon gene induction, the estrogen receptor α (ERα) binds to its cognate sequence (estrogen receptor-binding element, ERE) where it recruits the p400/Tip60 complex leading to loading of H2A.Z at the promoter and as consequence increased nucleosome positioning and finally to gene activation. At the same time, H2A.Z enrichment at the FBS is reduced [50]

The role of H2A.Z in nucleosome positioning was first described in yeast [81, 183]. Subsequently, Gévry and colleagues extended this observation to mammals when focusing on the promoter of the *trefoil factor 1* (*TFF1*) gene (Fig. 3, [50]). The positioning of the nucleosomes surrounding the ERα-binding element (ERE) at the *TFF1* gene promoter is stabilized upon activation of the pathway with estrogen; however, this effect is abolished by depletion of H2A.Z or p400 [50]. Cell-free studies also confirmed the role of H2A.Z in nucleosome positioning; however, the co-occurrence of both H2A.Z and H3.3 variants apparently does not play a major role when compared to the presence of H2A.Z only [178], suggesting that H2A.Z plays an important role in nucleosome positioning. This is also reflected in both the high instability of nucleosomes at the NFR and in the higher H2A.Z occupancy observed at dispersed promoters compared to the focused ones [163]. Additionally, there is a positive correlation between H2A.Z proximity to the TSS and gene expression level with genes highly expressed showing higher TSS proximal H2A.Z enrichment [80]. Together, this strongly supports a role for H2A.Z in nucleosome positioning. In future, it will be interesting to determine mechanistically whether PTMs and/or H2A.Z interactors play a role in the H2A.Z-mediated nucleosome positioning that in turn contributes to the enormous plasticity at promoters.

## H2A.Z and DNA methylation

Studies in *Arabidopsis thaliana* have shown that H2A.Z is excluded from sites enriched in DNA methylation and H2A.Z-occupied sites display low levels of DNA methylation [184]. This anti-correlation between H2A.Z occupancy and DNA methylation is recapitulated in other organisms [88, 185–188] and involves acetylation of H2A.Z [60], which is known to be the predominant mechanism in gene activation. Gene reactivation observed upon loss of DNA methylation, obtained via pharmacological inhibition or knockdown of DNA methyltransferases (DNMTs), is associated with a gain in H2A.Z occupancy [184, 189]. Similarly, increased H2A.Z occupancy, obtained via LoF of *ANP32E* that removes H2A.Z from chromatin [130], leads to reduced DNA methylation [188], while the opposite is observed upon gain-of-function (GoF) of *ANP32E*, LoF of the H2A.Z loading machinery or depletion of H2A.Z itself [88, 184, 188]. Notably, this mechanism may involve also another histone variant: macroH2A. In fact, it reversely correlates with H2A.Z occupancy but positively correlates with DNA methylation and gene silencing [190]. However, in contrast to the study of Yang and colleagues [189], Barzily-Rokni and colleagues do not observe gain in H2A.Z occupancy upon pharmacological inhibition of DNMTs alone or combined with macroH2A depletion even if gene expression is re-established [190]. One possibility to explain this discrepancy is represented by the different concentration of 5-azacytidine used in these studies [189, 190].

## Our current model for H2A.Z in gene regulation

The AR and ER systems represent good examples to explain the function of H2A.Z in gene transcription (Fig. 3). In the AR system, the *PSA* gene can be considered as the prototype of this pathway (Fig. 3a): In the absence of androgen (OFF state), H2A.Z is loaded by the SRCAP [119] and/or p400/Tip60 [122] complexes. In this repressed configuration, H2A.Z is monoubiquitinated at both enhancers and promoters [51] potentially by RING1B [58, 145]. Upon androgen stimulation (ON), H2A.Z is deubiquitinated by USP10 and its occupancy decreases [51, 52]. Of note, H2A.Zac correlates with AR induction [59, 122] and similarly, the occupancy of the p400/Tip60 complex increases upon AR induction [122]. The recruitment of the p400/Tip60 complex is mediated by its MRG15 subunit which recognizes H3K4 methylation states [122] while SRCAP has been shown to interact with AR [119]. In the case of the estrogen signaling cascade, we focus on the case of the *TFF1* locus (Fig. 3b): In the OFF state, forkhead box protein A1 (FoxA1) binds to a distal enhancer (FoxA1-binding site, FBS) of the *TFF1* locus where it recruits the p400/Tip60 complex supporting H2A.Z loading [50]. Lack of H2A.Z at the *TFF1* promoter, leads to a poorly defined nucleosome occupancy in the repressed/poised state (OFF, [50]). Upon activation of the pathway, the p400/Tip60 complex is recruited at the *TFF1* promoter by ERα which binds to its cognate sequences (ERE). At the *TFF1* promoter, the p400/Tip60 complex loads H2A.Z leading to a better-defined nucleosome positioning. At the same time, H2A.Z occupancy decreases at the FoxA1-bound distal enhancer [50].

From the above, some general rules for H2A.Z in gene regulation can be postulated: At genes that are poised/repressed (OFF), repressive marks of H2A.Z are found and as consequence its LoF leads to upregulation. At genes that are active, activating PTMs of H2A.Z, such as H2A.Zac, are found and as consequence H2A.Z LoF leads to downregulation.

In a repressed (OFF) or poised state, the H2A.Z deposition machinery is recruited by TFs and/or histone modifications to chromatin. This recruitment can be transient but still allows an exchange of H2A with H2A.Z. In the OFF state, H2A.Z is deacetylated by the deacetylation machinery and ubiquitinated on its C-terminus by RING1B. Upon gene activation (ON), additional TFs and/or histone modifications lead to the recruitment of the loading/acetylation/deubiquitination machinery. This triggers H2A.Z acetylation and deubiquitination, finally leading to transcriptional activation.

Still a number of open questions remain there. For example, the specificity of the p400/Tip60 and SRCAP complexes awaits to be determined in higher eukaryotes. Furthermore, there may be several p400-containing complexes that may act at different stages of gene transcription, the one without the acetyltransferase function and the other with Tip60 present. It is possible that these two complexes act in a stepwise fashion, one after the other. It is also possible that the different complexes are preferentially found at either promoters or enhancer elements (as described above). In addition, the H2A.Z removing factor ANP32E is also found in complex with Ep400 and Tip60 [130] suggesting that deposition, acetylation and removal of H2A.Z could be coupled in a stepwise manner. Chromatin-IP (ChIP) experiments with the relevant antibodies in dynamically regulated systems will answer such questions.

## H2A.Z in diseases

The histone variant H2A.Z, its PTMs and interacting proteins have been linked to several diseases, most notably cancer. H2A.Z expression is upregulated in metastatic melanoma [23], breast [191, 192], prostate [52, 59, 193], colorectal [194], liver [195, 196], bladder [74] and lung [126] cancer. Similarly, H2A.Z protein levels are elevated during cardiac hypertrophy [197] but decreased in diseased vascular tissues [161].

In prostate cancer, H2A.Zac has a pro-oncogenic role. It promotes activation of oncogenes and repression of tumor suppressor genes [60]. Regarding genomic occupancy, H2A.Zac increases at the TSS of oncogenes, while it decreases at the TSS of tumor suppressor genes [59, 60] and its genomic redistribution in prostate cancer leads to the activation of AR-associated neo-enhancers [59]. It must be marked that in metastatic melanoma both H2A.Z isoforms, H2A.Z.1 and H2A.Z.2, are upregulated; however, only the depletion of the H2A.Z.2 but not H2A.Z.1 leads to reduced proliferation [22]. In contrast to that, *H2AFZ* but not *H2AFV* is overexpressed in liver cancer and its knockdown results in reduced proliferation and inhibits the cancer cells’ metastatic potential [196]. These data support the notion that the different H2A.Z isoforms, which differ in only three amino acids, have distinct roles in the development of different tumor types.

While the data discussed so far highlight the upregulation of H2A.Z in cancer, additional mechanisms of H2A.Z deregulation may involve aberrant expression of the machineries involved in H2A.Z modifications and/or chromatin deposition/removal. For example, the methyltransferase SMYD3, which is upregulated in several cancer types, promotes proliferation of breast cancer cells and tumorigenesis [149]. This is achieved because SMYD3 supports H2A.Z methylation, which is required to activate the expression of the cyclin A1-encoding (*CCNA1*) gene [149]. Similarly, Tip60 is downregulated in acute myeloid leukemia (AML) samples [198] and present with mono-allelic loss in lymphomas, head-and-neck and mammary carcinoma [199]. In line with that, Tip60 has a tumor suppressor function in colon [200]. However, whether decreased Tip60 expression has any impact on H2A.Z deposition or acetylation is unknown.

Finally, the enzymes involved in the H2A.Z deposition can also be useful therapeutic targets, for example knockdown of SRCAP reduces cell proliferation of prostate cancer cells [119].

## The CRISPR/Cas9 system as a tool to deplete H2A.Z

**Fig. 4:**
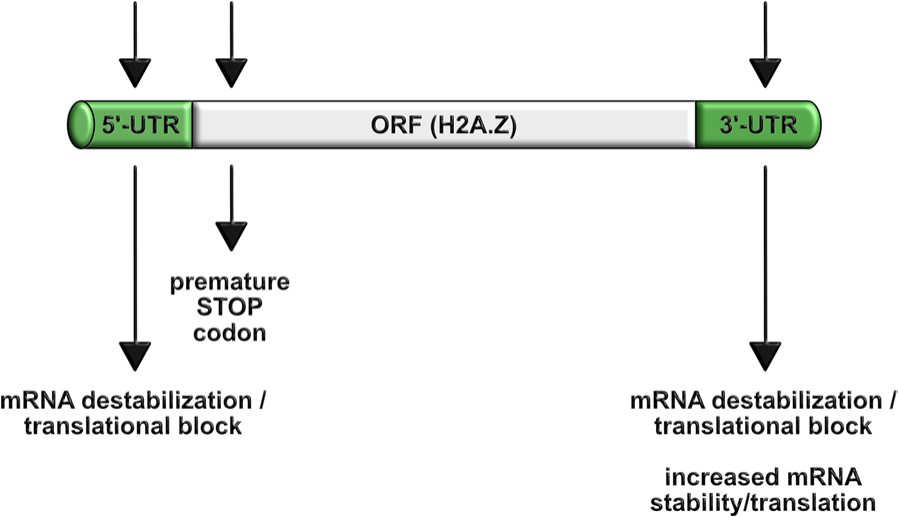
Summary of the different possible strategies that can be employed to deplete a histone gene via the CRISPR/Cas9 technology. A protein-coding gene can be targeted on its 5′-UTR leading to mRNA destabilization or preventing its translation. Alternatively, the ORF can be targeted, leading to the formation of a premature STOP codon. Finally, targeting of the 3′-UTR can lead to mRNA destabilization or to a translational block but it can also increase the mRNA stability or the translation efficiency. *UTR* untranslated region, *ORF* open reading frame

Histone variants emerged as essential regulators of embryonic development as their knockout is frequently lethal. Thus, obtaining even a successful knockdown in tissue culture cells is also a daunting task. We have recently used the CRISPR/Cas9 technology [201] to successfully (and also surprisingly for us) deplete the histone variant H2A.Z in a mouse T cell line [57]. Usually, the CRISPR/Cas9 technology is targeted to the 5′-end of open reading frames (ORF) of coding genes introducing mutations that lead to the generation of premature STOP codons (Fig. 4, [201]). We used this CRISPR/Cas9 system to target the 5′-UTR (untranslated) region of both *H2AFZ* and *H2AFV* genes [57]. In the literature, there are few examples of targeting UTRs: The 5′-UTR of the *Bcl2* gene in human cells has been targeted [202] and the 3′-UTR of the *Tyr* gene was targeted to study albinism in rabbits [203]. In another study, the 3′UTR of different chemokine genes was modified [204]. The authors observed differential effects when targeting the 3′-UTRs: while *CXCL3*, *CXCL10*, *CXCL11*, *CCL3*, *CCL4* and *CCL7* are upregulated, *CXCL1*, *CXCL6* and *CXCL8* are downregulated [204], suggesting that 3′UTRs may contain negative-regulating elements and that the CRISPR/Cas9 system can also be used to increase mRNAs abundance. Similarly, CRISPR/Cas9 targeting of the 3′UTR of the *PD*-*L1* gene leads to its overexpression [205]. Furthermore, the CRISPR/Cas9 system was used to edit the 3′UTR of *Cebpg* gene to prevent the mTOR (mammalian target of rapamycin)-mediated alternative polyadenylation [206]. Previously, zinc-finger nucleases (ZNFs) [207] were used to target 3′-UTRs in human cells [208]. Of note, most recently, the CRISPR/Cas9 technology was also used to deplete the histone variant H3.3B. However, in this case the authors targeted the coding sequence of the gene [209].

In summary (Fig. 4), the CRISPR/Cas9 technology can be used to target the 5′UTR or 3′UTR leading to deregulation of the target by promoting mRNA destabilization or translational block. However, targeting the 3′UTR can also be used to upregulate gene expression by increasing mRNA stability or translation. In our view, it is tempting to speculate that other histone variants or even canonical histones could also be targeted (or tagged/mutated/replaced) in this way as well as, the loading and removal machineries involved in the chromatin regulation of histone variants.

## Conclusions and perspective

We propose that the plastic behavior of H2A.Z at regulatory regions is ideally suited to buffer phenotypic noise by modulating transcriptional efficiency, both repressive and activating. Mechanistically, high levels of unmodified H2A.Z and ubiquitination of H2A.Z may serve as a roadblock for transcription; acetylation of H2A.Z and subsequent removal of H2A.Z may enhance transcription rate and/or help recruiting RNAPII or other activating complexes.

In the future, new tools such as highly specific antibodies against single-modified H2A.Z residues are needed to characterize the function of H2A.Z in development and in pathological settings. Since the current antibodies against H2A.Z (unmodified and pan-acetyl-H2A.Z) are exquisitely specific, there is a high chance that more specific reagents will have a major impact on chromatin and transcription research.

## Abbreviations

AhR: aryl hydrocarbon receptor
AML: acute myeloid leukemia
AP-1: activator protein-1
AR: androgen receptor
ARE: androgen receptor-binding element
BAH: domain bromo-adjacent homology domain
bPPI-seq: bait protein–protein interaction-sequencing
BPTF: bromodomain and PHD finger-containing transcription factor
BRD2: bromodomain-containing protein 2
*CCNA1*: Cyclin A1
ChIP: Chromatin-IP
DNMTs: DNA methyltransferases
DSBs: double-strand breaks
ELM2: Egl-27 and MTA1 homology 2 domain
EMT: epithelial-to-mesenchymal transition
ER: estrogen receptor
ERα: estrogen receptor alpha
ERE: estrogen receptor-binding element
eRNA: enhancer RNA
FACT: facilitates chromatin transcription
FBS: FoxA1-binding site
FoxA2: forkhead box protein A111
FoxA2: forkhead box protein A2
FRAP: fluorescence recovery after photobleaching
GoF: gain of function
GR: glucocorticoid receptor
GTF: general transcription factor
H2A.Zac: H2A.Z acetylation
H2A.Zsu: H2A.Z SUMOylation
H2A.Zub: H2A.Z ubiquitination
H3K4me1: H3 lysine 4 monomethylation
H3K4me3: H3 lysine 4 trimethylation
H3K14ac: H3 lysine 14 acetylation
H3K27ac: H3 lysine 27 acetylation
H3K122suc: H3 lysine 122 succinylation
HAT: histone acetyltransferase
HDAC: histone deacetylase
HR: homologous recombination
iFRAP: inverse fluorescence recovery after photobleaching
ISGF3: interferon-stimulated gene factor complex 3
KLK2: kallikrein-like 2
KMT: lysine methyltransferase
LoF: loss of function
MAGE: melanoma antigen-encoding gene
mESCs: mouse embryonic stem cells
MNase: Micrococcal nuclease
MNase-IP: immunoprecipitation of H2A.Z-containing mononucleosomes obtained via MNase digestion of chromatin
MS: mass spectrometry
mTOR: mammalian target of rapamycin
n.d.: not determined
ND: not defined
NDR: nucleosome-depleted region
NFR: nucleosome-free region
NuRD: nucleosome remodeling and deacetylase
NuRF: nucleosome remodeling factor
ORF: open reading frames
*Pf*GCN5: *Plasmodium falciparum* GCN5
PHD: plant homeodomain
PIC: pre-initiation complex
PSA: prostate-specific antigen
PTMs: posttranslational modifications
RARγ: retinoic acid receptor γ
RNAPII: RNA polymerase II
SANT domain: Swi3 Ada2 N-Cor and TFIIIB domain
SAS: something about silencing
SRCAP: SNF2-related CREBBP activator protein
SUMO: small ubiquitin-like modifier
TBP: TATA-binding protein
TF: transcription factor
TFBS: transcription factor binding site
TFF1: trefoil factor 1
TSA: trichostatin-A
TSS: transcription start site
USP10: ubiquitin-specific protease 10
UTR: untranslated
UVA: ultraviolet A
XCI: X-chromosome inactivation
ZNFs: zinc-finger nucleases
